# Spectrometric prediction of wood basic density by comparison of different grain angles and variable selection methods

**DOI:** 10.1186/s13007-021-00739-0

**Published:** 2021-03-31

**Authors:** Yanjie Li, Wenjian Liu, Ruishu Cao, Zifeng Tan, Jun Liu, Jingmin Jiang

**Affiliations:** 1 Research Institute of Subtropical Forestry, Chinese Academy of Forestry, Hangzhou, 311400 Zhejiang People’s Republic of China; 2 Institute of Horticulture Science, Massey University, Private Bag 11222, Palmerston North, 4442 New Zealand

**Keywords:** Wood properties, Wood basic density, Grain angle, Variable selection, NIR

## Abstract

**Background:**

Wood basic density (WBD) is one of the most crucial wood property in tree and mainly determined the end use of wood for industry. However, the measurement WBD is time- and cost-consuming, which an alternatively fast and no-destructive measurement is needed. In this study, capability of NIR spectroscopy combined with partial least squares regression (PLSR) to quantify the WBD were examined in multiple wood species. To obtain more accurate and robust prediction models, the grain angle (0° (transverse surface), 45°, 90° (radial surface)) influence on the collection of solid wood spectra and a comparison of found variable selection methods for NIR spectral variables optimization were conducted, including significant Multivariate Correlation (sMC), Regularized elimination procedure (Rep), Iterative predictor weighting (Ipw) and Genetic algorithm (Ga). Models made by random calibration data selection were conducted 200 times performance evaluation.

**Results:**

These results indicate that 90° angle models display relatively highest efficiency than other angle models, mixed angle model yield a satisfied WBD prediction results as well and could reduce the influence of grain angle. Rep method shows a higher efficiency than other methods which could eliminate the uninformative variables and enhance the predictive performance of 90° angle and mix angle models.

**Conclusions:**

This study is potentially shown that the WBD (g/cm^3^) on solid wood across grain angles and varies wood species could be measured in a rapid and efficient way using NIR technology. Combined with the PLSR model, our methodology could serve as a tool for wood properties breeding and silviculture study.

## Background

WBD (g/cm^3^), which is defined as the ratio of its oven-dry mass (at 0% moisture) to green volume (water- saturated wood volume), is a critical wood property that highly associate with other wood properties [1] for lots of industrial applications [2]. For instance, WBD could significantly influence the pulp yield, shrinkage and swelling behavior of wood [3]. In addition, WBD could efficiently reduce the mortalities caused by broken stems and uprooted trees in bad weather [4]. It also has been reported that WBD plays an important role in the estimation of carbon stocks from tree stems and biomass [5]. WBD must be high and uniform for using in a wide range of industry. However, WBD usually shows a considerable variation with and within trees of the same species. The radially from pith to bark, vertically from bottom to top within the stem and different tree sections (roots, stem and branches) have large WBD variations [6, 7]. Genetic breeding program could be an efficiently tool to reduce these variations [8, 9].

Standard methods to measure WBD have high levels of precision [10] but are time- and cost-consuming which could limit the estimation of WBD cycling when a large number of samples need to be measured. Therefore, it is required to find out a fast and low cost method to replace these methods for the WBD determination.

Near-infrared spectroscopy (NIRS) is an efficient and high-throughput technique that has been used in chemical component analysis in many fields. It is a promising and reliable method for the assessment of large samples [11–16]. It basically relies on the variation in the adsorption spectra, such as the vibration, stretching and bending of molecular bonds. Special bonds, including C–H, N–H and O–H bonds [17], will interact with the specific wavelengths in the NIR spectroscopy. A stable and uniform illumination source of NIR spectra combined with consistently collected samples could provide a better platform for organic chemicals [18]. To obtain a better prediction result, multivariate methods such as partial least squares regression (PLSR) [19] will be used by pairing the NIR spectra and independent chemical measurements together to calibrate a high accuracy prediction model. The satisfying model will then be applied to unknown samples and the spectra data will be used for independent chemical prediction. Recent researches have shown that WBD and other wood properties are predictable by using laboratory near infrared spectrophotometry in different species [20–22]. However, little is known about the NIR spectroscopy utilization in analysing the variation of WBD in different tree species under different grain angle conditions [23, 24].

The way of NIR spectra collection on the wood samples could be a significant influence on the accuracy of model calibration, and the models that based on the NIR spectra of wood powder have been reported that yield better accuracy than the models that based on spectra of solid wood samples [25]. However, it is also a laborious step to grain wood into powder which is not suitable for larger samples measurements. Alternatively, it will be more efficient to predict WBD from solid wood samples.

The variation of grain angle in radial and vertical direction of solid wood could influence the spectra information for model calibration [26]. It has been reported that the grain angle could influence the EC prediction of *Eucalyptus bosistoana* using NIR spectra and this influence could be reduced by using of EPO algorithm [22]. However, little is known about the grain angle influence on WBD across many tree species. In addition, the spectra bands, which contain massive overtones and combinations of vibrations information from hydrogen-containing groups (C–H, O–H, and N–H) in wood samples [27], usually highly-overlapping and contain many collinearity and irrelevant information resulting in difficult to directly distinct the interested wood properties and highly influence the robustness and reliability of model calibration [28]. Despite the complicated band assignment for different chemical compositions, Several pre-processing methods, such as SNV and derivatives, could efficiently reduce these bands influence before model calibration [29, 30]. Additionally, it has been reported that the use of importance feature selection from the spectra instead of using the full length of spectra to calibrate model could yield a robust and highly accurate prediction result and efficiently reduce the redundant noise and band information [31, 32]. There are many mathematic variable selection algorithms combine with chemometric statistics that have been used to improve the performance of the model by eliminating the irrelevant variables [33, 34], such as a significant Multivariate Correlation (sMC) algorithm [35], Regularized elimination procedure (Rep) algorithm [36], Iterative predictor weighting (Ipw) [37] and Genetic algorithm (Ga) [38]. However, the comparison of different variable selection algorithms combines with PLSR method for quantitative prediction of multispecies wood, especially for WBD which has been less reached.

Therefore, (1) we tested the capacity of reflectance spectroscopy to characterize the WBD in various of hardwoods tree species using PLSR model, and (2) we focused on the comparison of different grain angle models for the better prediction of WBD, (3) we compared the performance of four variable selection methods, including sMC, Ipw, Rep and Ga, for improving the predictive performance of PLS calibrations and to identify the most important wavelength related to WBD. More importantly, (4) we tested the possibility of using a mixed angle (global) calibration models with relevant informative variables for a fast WBD prediction.

## Results

### Spectra information

**Fig. 1:**
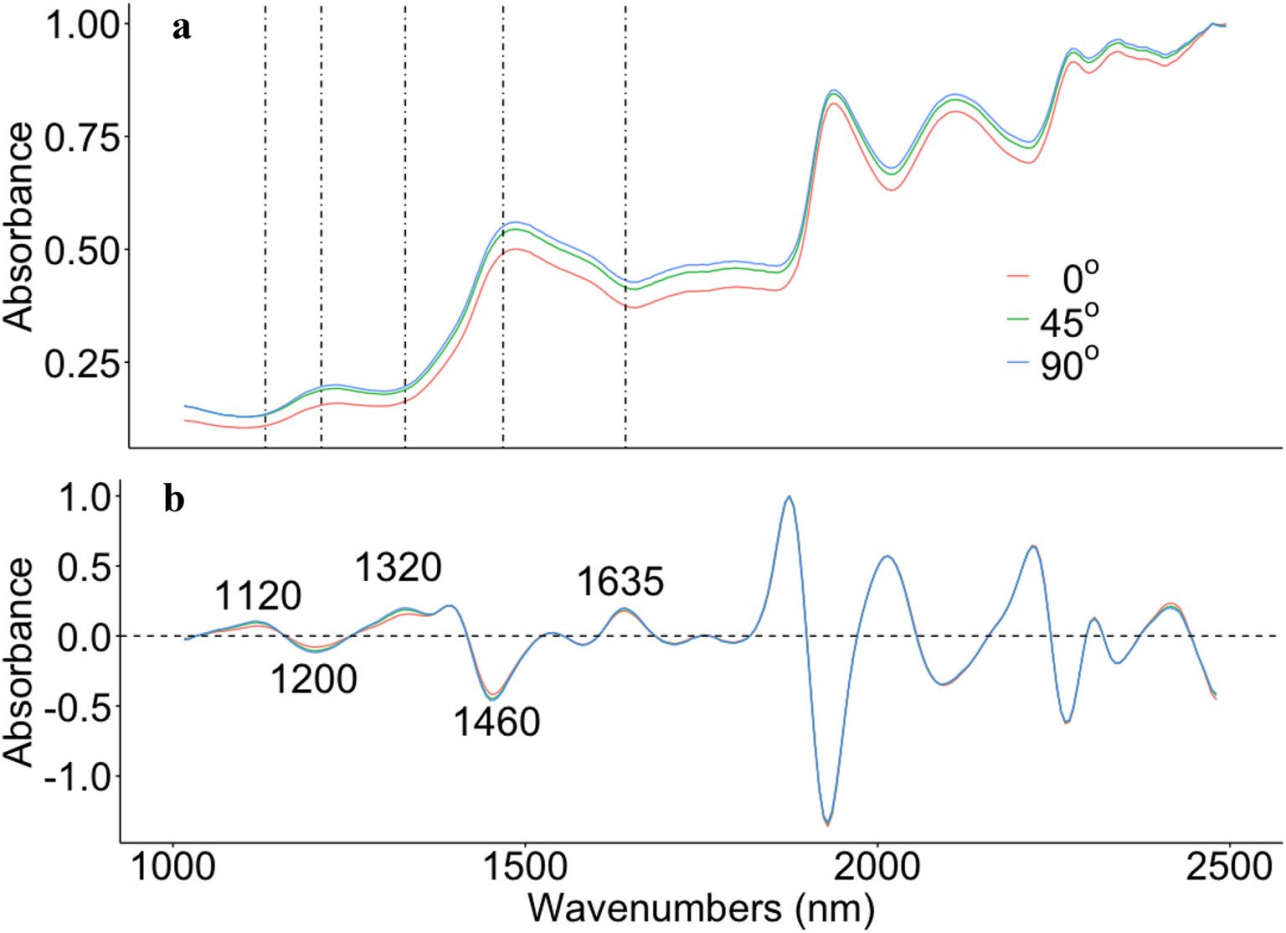
Orignal (Raw)-NIR (**a**) and 2nd derivate-NIR (**b**) spectra of three grain angle directions from wood cores of varies tree species. Dot line: the position same as in **b**

The averaged three grain angles original (none pre-processing) and 2nd derivate spectra were ploted in Fig. 1. It is clearly shown that the original spectra of three angles have similar signal curve and hard to identify with the naked eyes. However, spectra start to show differently in four different bands between three angles after pre-processed by 2nd derivate. The 45° and 90° degree are shown similar curve to each other but both have slightly difference from 0° degree.

### Prediction of WBD using full length NIR spectra

**Fig. 2:**
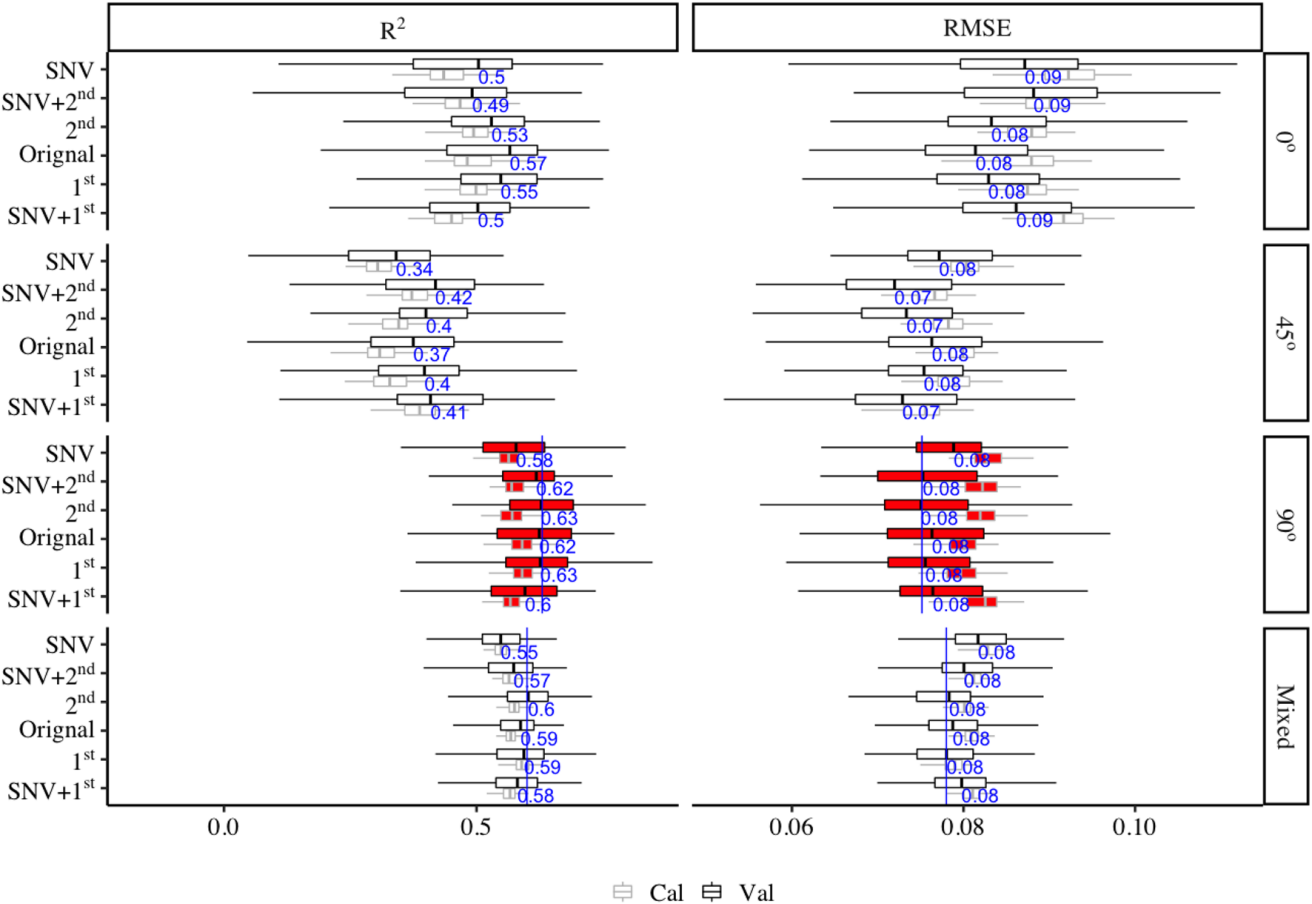
Distribution (95% confidence intervals) of calibration and validation statistics from 200 simulations of models predicting WBD from 0°, 45°, 90° angles and the mixed angle of multiple tree species using full length NIR spectra. Each model permutation included 80% of the data for internal calibration and the remaining 20% for validation. The blue vertical line represents the highest R^2^ and lowest RMSE (g/cm^3^) value, The black vertical line in each box represents median value, the red colour box represents the 90° angles model

**Fig. 3:**
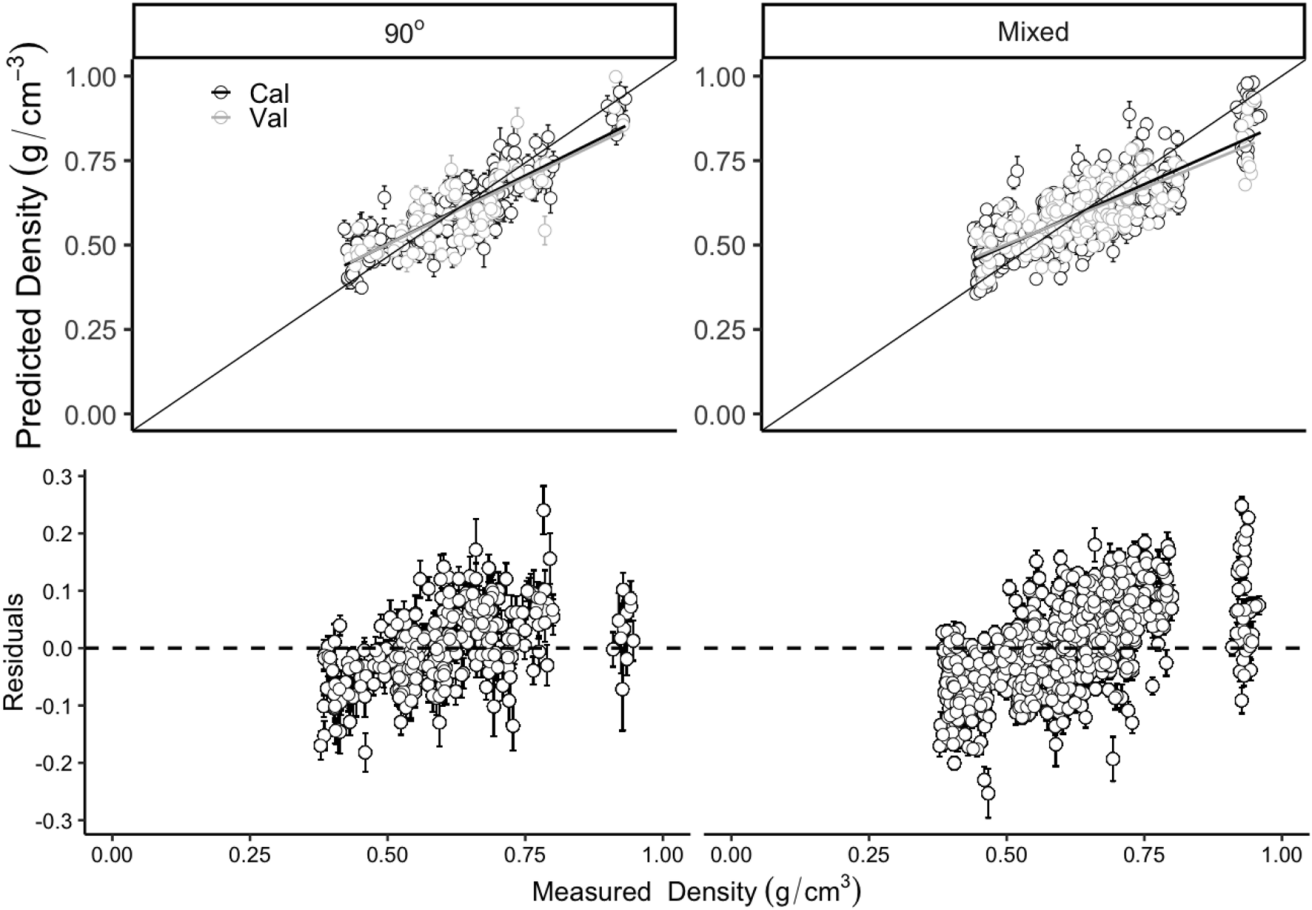
Measured and predicted WBD (g/cm^3^) (top two) and Residuals plotted against measured Density (g/cm^3^) (bottom two) in the 90° angles and mixed angle model of multiple tree species using full length NIR spectra. Error bars for predicted values represent the standard deviations obtained from the 200 simulated models

The WBD shown a wide variance from 0.39 to 0.93 with a mean of 0.61 across different wood species and could be efficiently predicted with PLSR models using full length NIR spectra on three grain angles and mixed angle models. Despite to all of the six different NIR spectra processing methods (including no processing), 90° angle model produced the highest mean ${\text{R}}_{\text{Cal}}^{ 2}$ (calibration) of 0.58 (ranged from 0.46 to 0.69) and ${\text{R}}_{\text{Val}}^{ 2}$ (Validation) of 0.60 (ranged from 0.35 to 0.85) respectively, followed by mixed models (mean ${\text{R}}_{\text{Cal}}^{ 2}$ and ${\text{R}}_{\text{Val}}^{ 2}$ were 0.57 (range: 0.51–0.63) and 0.58 (range: 0.48–0.74) respectively), 0° angle models (mean ${\text{R}}_{\text{Cal}}^{ 2}$ and ${\text{R}}_{\text{Val}}^{ 2}$ were 0.47 (range: 0.33–0.63) and 0.50 (range: 0.05–0.76) respectively) and 45° angle models (mean ${\text{R}}_{\text{Cal}}^{ 2}$ and ${\text{R}}_{\text{Val}}^{ 2}$ were 0.34 (range: 0.21–0.48) and 0.38 (range: 0.05–0.69) respectively). Six different NIR spectra processing methods (including no processing) shown different effects on the precision of WBD. PLSR models from 2nd normalised spectra showed the smallest errors in WBD prediction from 90° and mixed angle models with the mean ${\text{R}}_{\text{Val}}^{ 2}$ of 0.63 (ranged from 0.35 to 0.84) and of 0.60 (ranged from 0.43 to 0.73), the mean RMSE_Val_ of 0.08 g/cm^3^ (range: 0.05–0.09 g/cm^3^) and of 0.08 g/cm^3^ (range: 0.07–0.10 g/cm^3^) respectively (Fig. 2). Very small prediction error was obtained from the 100 simulated models for both the 90° angle model and the mixed angle model (Fig. 3 top two). Residual plots have shown that both the 90° angle model and the mixed angle model tend to underestimate in the low WBD values and overestimate when WBD is high (Fig. 3 bottom two).

### Comparison of four variable selection methods

**Fig. 4:**
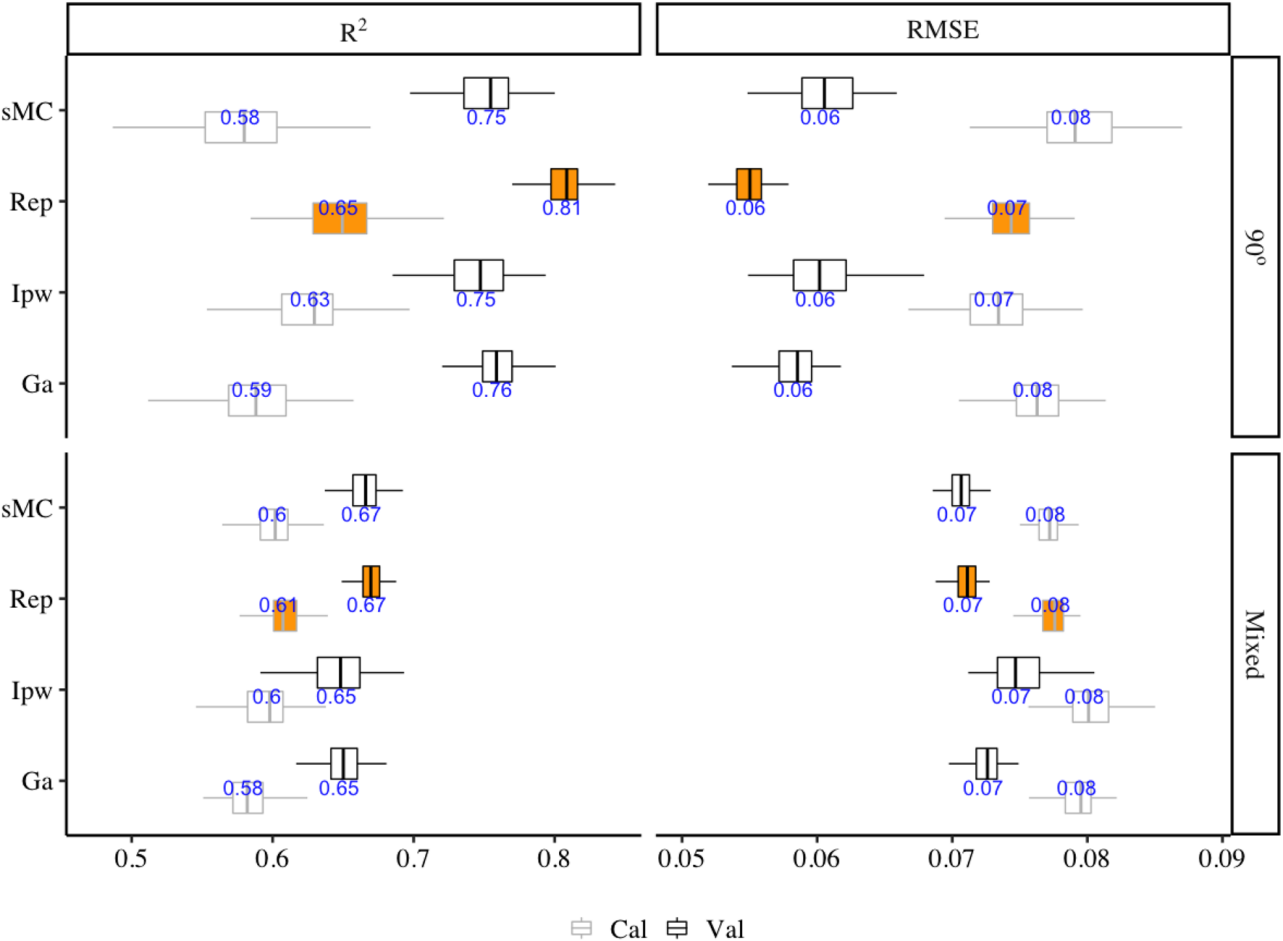
Distribution (95% confidence intervals) of calibration and validation statistics from 100 simulations of models predicting WBD (g/cm^3^) in the 90° angles and mixed angle model of multiple tree species using four different variable selection methods on NIR spectra. Each model permutation included 80% of the data for internal calibration and the remaining 20% for validation. The black vertical line represents median value; the orange colour box represents the Rep variable selection methods

**Fig. 5:**
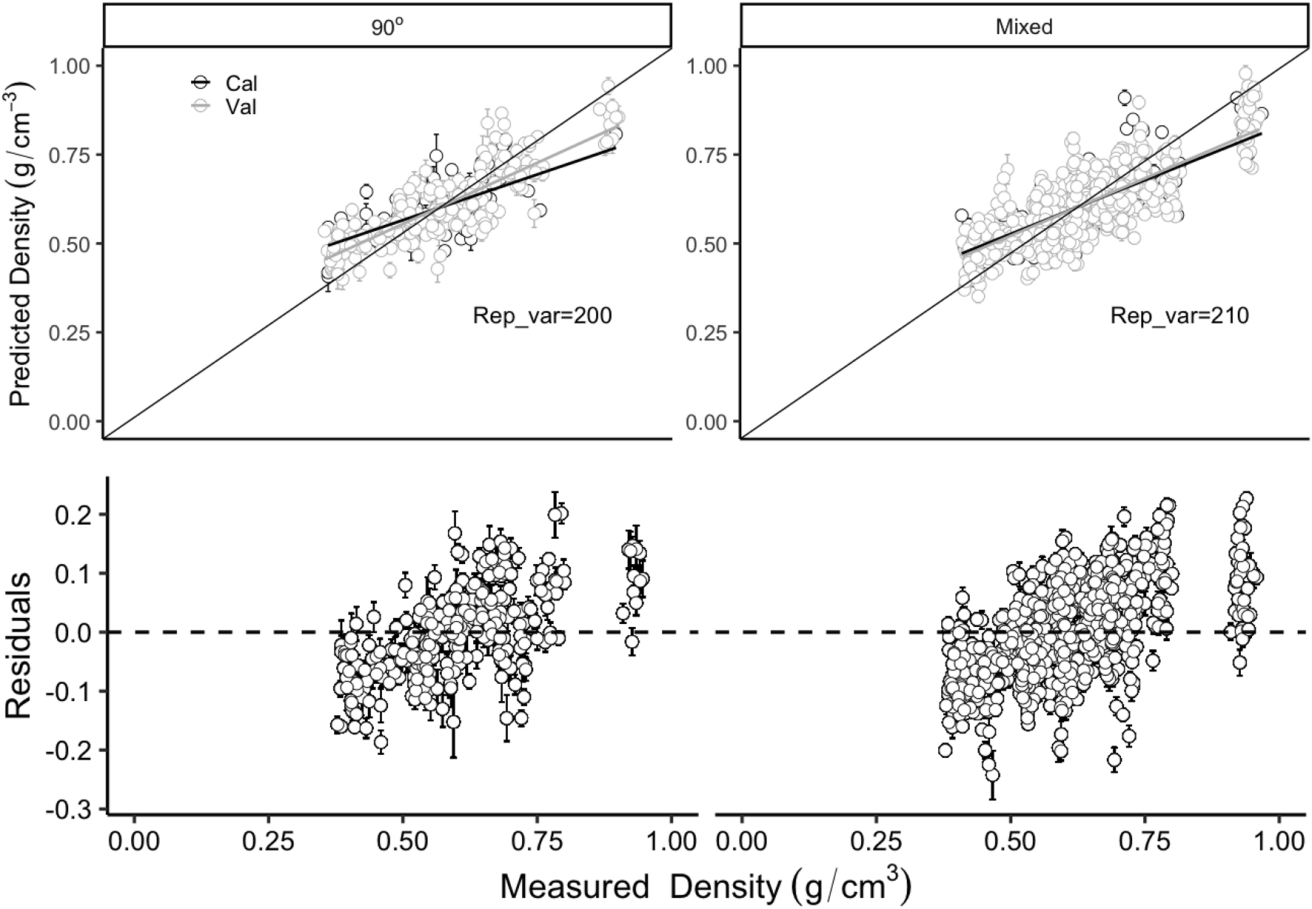
Measured and predicted WBD (g/cm^3^) (top two) and Residuals plotted against measured Density (g/cm^3^) (bottom two) in the 90° angles and mixed angle model of multiple tree species using the Rep-selected NIR spectra. Rep_var: variables selected by Rep algorithm; Error bars for predicted values represent the standard deviations obtained from the 200 simulated models

The performance of 90° angles and mixed angle PLSR models using four different variable selection methods were plotted in Fig. 4. The prediction accuracy of 90° angle model has been improved much better than the mixed angle model by these four different variable selection methods. The highest prediction model from 90° and mixed angle were found by using of Rep-selected NIR spectra variables with the mean ${\text{R}}_{\text{Val}}^{ 2}$ of 0.81 (ranged from 0.77 to 0.84) and of 0.67 (ranged from 0.64 to 0.70), the mean RMSE_Val_ of 0.07 g/cm^3^ (range: 0.071–0.074 g/cm^3^) and of 0.05 g/cm^3^ (range: 0.05–0.06 g/cm^3^) respectively, followed by Ga, sMC and Ipw algorithm which had a mean ${\text{R}}_{\text{Val}}^{ 2}$ of 0.76 (range: 0.70–0.80), 0.75 (range: 0.70–0.80),0.75 (range: 0.66–0.80), mean RMSE_Val_ of 0.058 g/cm^3^ (range: 0.05–0.06 g/cm^3^), 0.06 g/cm^3^ (range: 0.055–0.066 g/cm^3^), 0.06 g/cm^3^ (range: 0.05–0.07 g/cm^3^) in 90° angle model and the order has been changed into sMC, Ga and Ipw which had a mean ${\text{R}}_{\text{Val}}^{ 2}$ of 0.66 (range: 0.64–0.69), 0.65 (range: 0.62–0.68),0.64 (range: 0.55–0.69), mean RMSE_Val_ of 0.068 g/cm^3^ (range: 0.071–0.071 g/cm^3^), 0.073 g/cm^3^ (range: 0.069–0.075 g/cm^3^), 0.075 g/cm^3^ (range: 0.071–0.083 g/cm^3^) in mixed angle models respectively. Mixed tissue models showed a promising WBD (g/cm^3^) prediction result. Similar to the PLSR tissue models that use a full length NIR spectra, models are more variable on the validation set than the calibration set. 90° and mixed models showed similar error predictions after 200 simulation by using less spectra variables (200 and 210 respectively) (Fig. 5 top two). The residuals of all models that based on Rep selected variables showed similar results to the full-length spectra models with underestimate in the low WBD values and overestimate when WBD is high (Fig. 5 bottom two).

### Variable selection of the NIR spectra applied in WBD (g/cm^3^) prediction

**Fig. 6:**
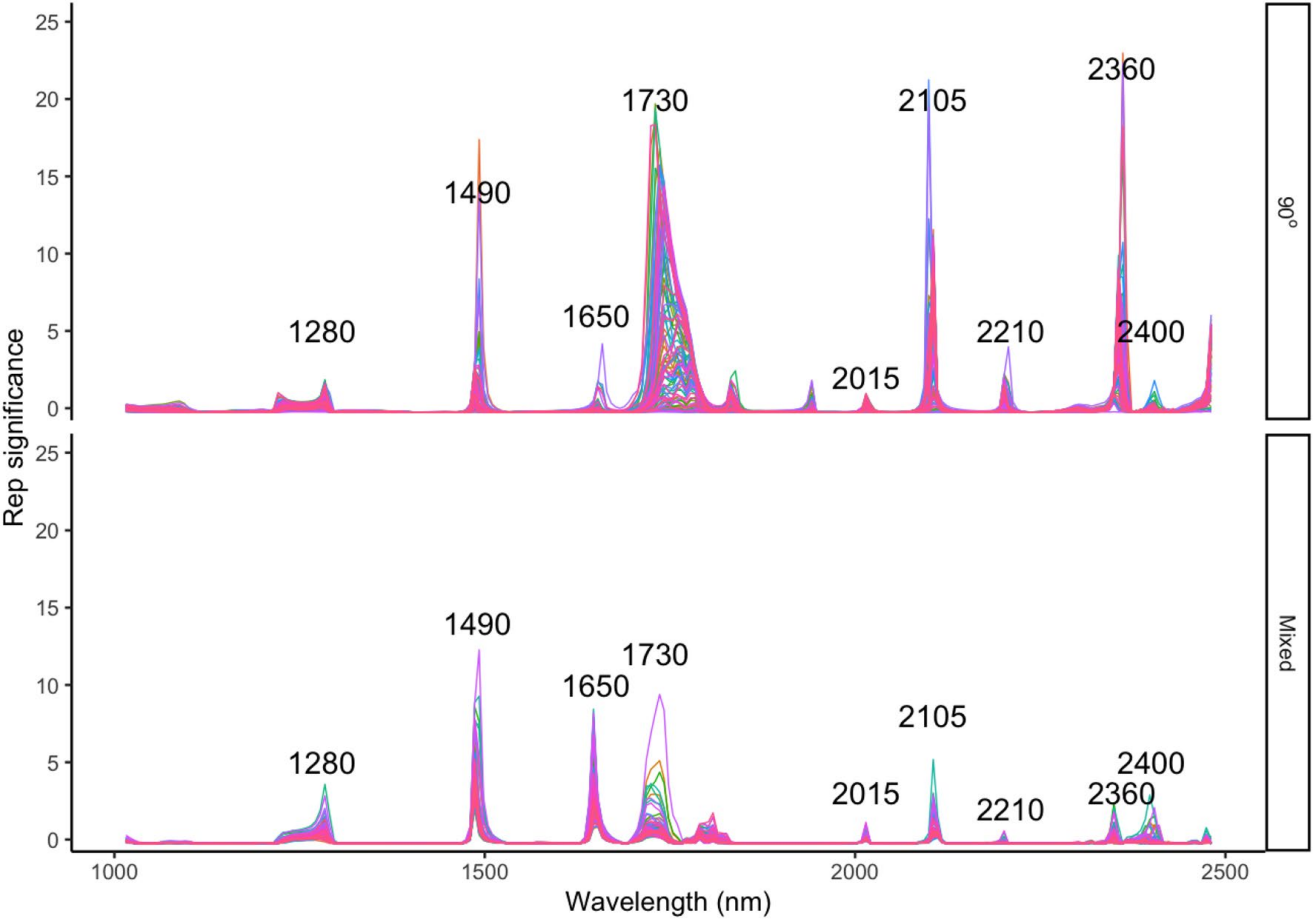
Influence of WBD (g/cm^3^) on NIR spectra in the 90° angles and mixed angle model that randomly conduct 200 times of multiple tree species and the variables selected by the Rep algorithm. Each line mean one time of modelling with Rep variable selection

Both the 90° angles and mixed angle models were conducted 200 times with Rep variable selection to find out the most important regions that highly related to WBD (g/cm^3^), the results have been showed in Fig. 6. Nine significantly important regions, i.e., 1280, 1490, 1650, 1730, 2015, 2105, 2210, 2360 and 2400 nm were found that have a great influence on the performance of prediction models even after conducing 200 times. In 90° angles model, the band at 2360 nm has been considered as the most import region than other bands, followed by the 2105, 1730, 1490 and others. The bands at 2015 nm is the lowest importance bands compared to the other 9 bands. In contrast, the mixed model considered the band at 1490 nm is the most important than other bands, followed by 1730 and 1650 nm, which mostly located in the region between 1400 and 1700 nm. The bands at 2360 and 2105 nm in Mixed angle model do not present as important as in 90° angles model.

## Discussion

In this research, the WBD (g/cm^3^) shows a high variation in different types of wood species. The variation of response characteristics highly affect the model accuracy. Less variation of the response characteristics will result in a relative lower quality of model prediction [19].

With the high range mean of ${\text{R}}_{\text{Val}}^{ 2}$ (0.77–0.84) and (0.64 to 0.70) and the low range mean of RMSE_Val_ (0.071–0.074 g/cm^3^) and (0.05–0.06 g/cm^3^) that obtained from the 90° angles and mixed angle models respectively, our results clearly shows that the WBD in different types of wood species can be reasonably and accurately predicted using NIR reflectance spectroscopy. Furthermore, a mixed grain angle model can efficiently predict the WBD from different grain angle spectra.

The mean ${\text{R}}_{\text{Val}}^{ 2}$ of 90° angles model (0.81, range: 0.77–0.84) in our results was mostly equivalent to the result reported in multiple wood chips (R^2^ = 0.89) [39] and Dahurian larch (R^2^ = 0.84), Japanese elm (R^2^ = 0.83) and Chinese white poplar (R^2^ = 0.84) [40] when using the VIS–NIR/NIR spectroscopy to predict WBD. The spectra from 90° angles (transverse) shown the best results compared to other angles for the prediction of WBD, the 45° and 0° angles showed similar lower prediction results than the 90° angle model. Supported et al. [41] and Schimleck et al. [42] who found that the transverse face yields better WBD and other wood properties prediction results than the radial face from many pine species. Suggested that use of the transverse surface for the better prediction of wood properties and avoid the grain angle influence, cause other angles like tangential face, which contains uncertain earlywood or latewood ring, could highly influence the spectrum collection [43]. In the contrary, we found that the mixed angle model could provide a reliable and promising result for the WBD prediction. A similar result has been found that the calibration models based on both radial and tangential surface spectra could yield a satisfy result and reduce the angle influences [44]. Mixed model methods also has been used in the prediction of other plant chemical components like NSC concentration in different tree tissues (root, stem, branch and leaf) of various tree species [15] and Total non-structural carbohydrate (TNC) concentration in both leaves and trunks [45]. Although the accuracy of mixed model in our study was slightly low compared to 90° angles model. However, it has given an additional unnecessary step of building a single tissue model. Our results strengthen that the NIR methods for the prediction of the WBD from different grain angle spectra is reliable and can be used as a surrogate for standard chemical analyses.

A robust methodology which was initially presented by Couture et al. [46] has been used in this study. Multiple permutations of the data allow estimating the data distribution and model stability. Furthermore, it could provide the prediction error based on the 200 times calibrations (see Figs. 3 and 5 error bar) which could be used for the model uncertainty test. The obvious model prediction errors and the stability between models prove that this iterative methodology is a useful algorithm for future model calibrations. Thus, we highly recommend to use this methodology for model calibrations and validations on NIR analysis.

The NIR spectra of samples contains not only useful chemical information but also many noises and irreverent information which may interfere with the model accuracy of the prediction. Therefore, a variable selection is very important to find out the most important wavelength which could contribute to minimizing the error for model calibrations and helping reduce the model processing time [47].

The comparisons of four variables selection methods shown that the Rep methods displayed the highest performance in the prediction of WBD and could select important variables that correlated greatly with WBD in the NIR range. It is different to the results reported by [48] who found that the successive projections algorithm combined with interval partial least squares which could efficiently yields a satisfy result for the prediction for WBD in *Mimosa tenuiflora* [Willd.] Poiret wood. The Rep_PLSR models yielded a better and promising result with only a small set of spectra variables (ranging from 200 to 210 among two models) (Fig. 5) compared to the full length spectra variables. In addition, Rep identified that the key wavelengths regions that highly correlated with the WBD located between 1200–1800 nm and 2000–2400 nm. Ten most important peaks among these regions were found, i.e., 1280, 1490, 1650, 1730, 2015, 2105, 2210, 2360 and 2400 nm. The bands around 1280, 2105 and 1730 are primarily attributed to the first overtone absorptions of CH groups in cellulose and hemicellulose. The band around 1726 nm is associated with the CH stretching of CH_2_-groups or lignin. The region around 1490 and 1650 nm was mainly related to the first overtone of O–H strength which may be dominated by the cellulose in woody samples [49]. Band appearing at 2210 nm is assigned to the band of C–H stretching vibration from lignin. The C–H deformation and stretching vibration of cellulose are indicated by spectral regions of 2360 and 2400 nm.

In NIR spectra, water has a wide absorbance region which could be a major influence on other chemical information causing spectra overlap. Min et al. [50] found that the regions of 1910 and 1938 nm which are highly related to water which may have a strong influence on plant properties prediction. Thus, in this study, these regions have been avoided in the mixed angle models.

The cost and time of traditional methods for the assessment of a large-scale wood property are consuming and will limit the process of reaching in forestry and wood product-related industries and also the understanding of variation wood properties in genetic level. Our fast and accurate measurement of WBD from different grain angels using NIR spectroscopy provide an advanced way for the study of wood quality and allow for large samples quick measurement.

## Conclusions

Our study have shown that the utility of near infrared spectroscopy combined with PLSR and variable selection methods successfully use NIR spectroscopy to characterize the WBD using various hardwoods species. 90° angles (transverse) model were present the best prediction for WBD, but the mixed model also yields a promising and reliable results which could reduce the grain angle influence. For the acquisition of accurate and robust spectra prediction model, a appropriate variable optimization is crucial and needed. However, different variable selection yield varies from prediction accuracy in the NIR prediction model. Our study shown that Rep methods displayed a higher accuracy for WBD prediction relatived to other methods. Methodologically, These results demonstrate the potential of variable-optimized NIR models for wood quality assessment in practical wood production.

## Materials and methods

### Sampling and WBD measurement

A considerable WBD variation among tree species is needed to get an accurate chemometric model [51]. As such, in the autumn of 2018, we collected 300 samples from thirty-three different tree species with varies ages in Miaoshangwu Mountains in Hangzhou, China (30° 05′ N, 120° 01′ E) (Table 1). Wood cores were collected by drilling into the tree stem with a 12 mm diameter drill at breast height. Each core sample was placed into a Kraft paper bag and immediately shipped to the laboratory for measurement of core fresh weight. Samples were dried to a constant weight in oven at 104 °C for measurement of core dry weight. The WBD of the wood was determined as the dry matter weight per unit volume of green wood. Three angles were marked on each core, including 0° (transverse surface), 45°, 90°(radial surface), based on the stem axis, the surface of each degree was sanded with a P80 grid sandpaper, and details description can be found in Li and Altaner [22].

**Table 1:** Wood species that selected for wood cores

Names	Number of cores
*Cunninghamia lanceolata*	6
*Cyclocarya paliurus*	8
*Fokienia hodginsii* (Dunn) Henry et Thomas	7
*Camptotheca acuminata*	5
*Liquidambar formosana*	5
*Cinnamomum camphora* (Linn) Presl	6
*Sapium sebiferum* (L.) Roxb	6
*Michelia maudiae* Dunn	6
*Elaeocarpus sylvestris*	6
*Kalopanax septemlobus* (Thunb.) Koidz	6
*Magnolia denudata*	6
*Tapiscia sinensis* Oliv.	7
*Pinus elliottii*	7
*Choerospondias axillaris*	7
*Magnolia macclurei*	7
*Diospyros montana* Roxb	8
*Parakmeria lotungensis* (Chun et C. Tsoong) Law	8
*Michelia chapensis*	8
*Pistacia chinensis*	8
*Dalbergia balansae*	9
*Vernicia fordii*	10
*Manglietia fordiana* Oliv.	10
*Zelkova serrata*	11
*Pinus taeda*	10
*Pseudotsuga gaussenii*	11
*Michelia foveolata* Merr. ex Dandy	12
*Michelia odora* (Chun)Noot. et B. L. Chen	12
*Photinia davidsoniae* Rehd. et Wils	13
*Phoebe chekiangensis*	15
*Magnolia liliflora* Desr	15
*Nyssa sinensis* Oliv	15
*Phoebe bournei* (Hemsl.) Yang	16
*Keteleeria fortunei*	14

### Spectral collection

All the NIR Reflectance spectra of dried wood cores in three-degree directions were collected every 10 mm along the core using a NIR spectrometer (LF-2500, Spectral evolution, USA) with a 5 mm diameter fiber-optical probe. Spectra were obtained with a range of 1100 to 2500 nm and a spectral resolution of 8 nm. Each spectrum point was scanned 32 times and averaged as the absorbance spectra (log 1/R, where R = reflectance), for each core, the spectra was averaged respectively in respect to each degree.

### Model calibration and validation

PLSR [19] models with leave-one-out cross-validation were generated to predict the WBD of wood core from three different grain angle directions (0°, 45°, 90°). PLSR holds the advantages of producing reliable coefficients, reducing the bias and estimate errors and using fewer PLSR components, all of which make it one of the most commonly used methods for chemometric analyses [52, 53]. Two types of pre-processing methods including stander normal variate (SNV), 1st and 2nd derivatives with a window size of 15 data points using Savitzky-Golay smoothing [54] and their combinations were compared in our study. SNV has been widely use for scatter correction of spectra data, while the derivatives can efficiently remove both additive and multiplicative effects in the spectra [55].

80% of the data set was selected for calibrations and the remaining 20% was used for validations. For each angle and pre-processing calibration, the model was conducted 200 times for the evaluation of performance [46]. The coefficient of determination (R^2^) and root-mean-square error (RMSE) derived from both the calibration (Cal) and validation (Val) were used to track the model performance. Four types of variable selection (sMC, Ipw, Rep and Ga) were used to find out the best performance of the PLSR models with small subset of spectral variables. Data analysis was conducted in R software (version 3.1.2) [56]. Some setup packages in R were used for this study, including the pls package [57] for PLSR and sMC-PLSR model performing and plsVarSel [36] for variables selection.

## Data Availability

Not applicable.
